# Supplementary material for: A Five Year Randomized Controlled Trial to Assess the Efficacy and Antibody Responses to a Commercial and Autogenous Vaccine for the Prevention of Infectious Bovine Keratoconjunctivitis
Source: Vaccines (Basel). 2022 Jun 9;10(6):916. doi: 10.3390/vaccines10060916 (PMC9228096; doi:10.3390/vaccines10060916)
Supplement: Supplementary file 1 [file vaccines-10-00916-s001.zip › vaccines-1722333-supplementary.pdf]

Supplementary Table S1: Calves enrolled in each treatment group for the years analyzed in this study.

	**Autogenous**	**Commercial**	**Sham**	Total
**2017**	68	73	71	212
**2019**	76	64	72	212
**2020**	85	85	78	248

Supplementary Table S2: Sequence of rPilA antigen and negative control scrambled peptide antigen used in this study.

Antigen	Amino Acid Sequence
Recombinant full length PilA (rPilA)	MNAQKGFTLIELMIVIAIIGILAAIALPAYQ DYISKSQTTRVVGELAAGKTAVDAALFEG KTPKLGKAANDTEEDIGLTTTGGTARSNL MSSVNIGGGAFATGAGTLEATLGNRANKD IAGAVITQSRDAEGVWTCTINGSAAPGWK SKFVPTGCKEH
Scrambled peptide	NLWGEANPNSGNGCSAQALSGLQPAQMT KVGDTKCSLT

Supplementary Table S3: Predicted probability of IBK development (Mean x 100%) for each combination of treatment, management group, and sex.

Phenotype combination	Estimate	Standard Error	Mean	SEM
**Autogenous Black Angus Heifer**	-1.7993	0.2993	0.1419	0.03646
**Autogenous Black Angus Bull**	-1.5722	0.2927	0.1719	0.04166
**Commercial Black Angus Heifer**	-1.5201	0.3058	0.1794	0.04502
**Autogenous Red Hybrid Heifer**	-1.4603	0.2177	0.1884	0.03328
**Sham Black Angus Heifer**	-1.4499	0.2987	0.1900	0.04598
**Commercial Black Angus Bull**	-1.293	0.2897	0.2154	0.04895
**Autogenous Red Hybrid Bull**	-1.2332	0.2201	0.2256	0.03845
**Sham Black Angus Bull**	-1.2227	0.2918	0.2275	0.05127
**Commercial Red Hybrid Heifer**	-1.1811	0.2162	0.2348	0.03884
**Autogenous Black Hybrid Heifer**	-1.1287	0.209	0.2444	0.0386
**Sham Red Hybrid Heifer**	-1.1109	0.2033	0.2477	0.03788
**Commercial Red Hybrid Bull**	-0.954	0.2053	0.2781	0.04122
**Autogenous Black Hybrid Bull**	-0.9016	0.2061	0.2887	0.04232
**Sham Red Hybrid Bull**	-0.8838	0.2055	0.2924	0.04251
**Commercial Black Hybrid Heifer**	-0.8495	0.2096	0.2995	0.04398
**Sham Black Hybrid Heifer**	-0.7793	0.2083	0.3145	0.0449
**Commercial Black Hybrid Bull**	-0.6224	0.1926	0.3492	0.04376
**Sham Black Hybrid Bull**	-0.5521	0.2049	0.3654	0.04751

Supplementary Table S4: Predicted probability of retreatment (Mean x 100%) for each combination of treatment, management group, and sex.

Phenotype combination	Estimate	Standard Error	Mean	SEM
**Autogenous Black Angus Heifer**	-1.7768	0.4526	0.0378	0.03725
**Commercial Black Angus Heifer**	-1.5583	0.4503	0.05958	0.05335
**Autogenous Black Angus Bull**	-1.4013	0.4335	0.08056	0.06479
**Sham Black Angus Heifer**	-1.278	0.4271	0.1006	0.0753
**Commercial Black Angus Bull**	-1.1829	0.4296	0.1184	0.08514
**Autogenous Red Hybrid Heifer**	-1.159	0.2726	0.1232	0.05556
**Commercial Red Hybrid Heifer**	-0.9405	0.2598	0.1735	0.06659
**Sham Black Angus Bull**	-0.9026	0.4091	0.1834	0.1086
**Autogenous Black Hybrid Heifer**	-0.8129	0.2488	0.2081	0.07134
**Autogenous Red Hybrid Bull**	-0.7835	0.2536	0.2167	0.07443
**Sham Red Hybrid Heifer**	-0.6602	0.2411	0.2546	0.07735
**Commercial Black Hybrid Heifer**	-0.5944	0.2185	0.2761	0.07306
**Commercial Red Hybrid Bull**	-0.5651	0.2368	0.286	0.08055
**Autogenous Black Hybrid Bull**	-0.4374	0.2391	0.3309	0.08667
**Sham Black Hybrid Heifer**	-0.3141	0.2303	0.3767	0.08747
**Sham Red Hybrid Bull**	-0.2848	0.2234	0.3879	0.0856
**Commercial Black Hybrid Bull**	-0.219	0.204	0.4133	0.07944
**Sham Black Hybrid Bull**	0.06134	0.2238	0.5245	0.08912

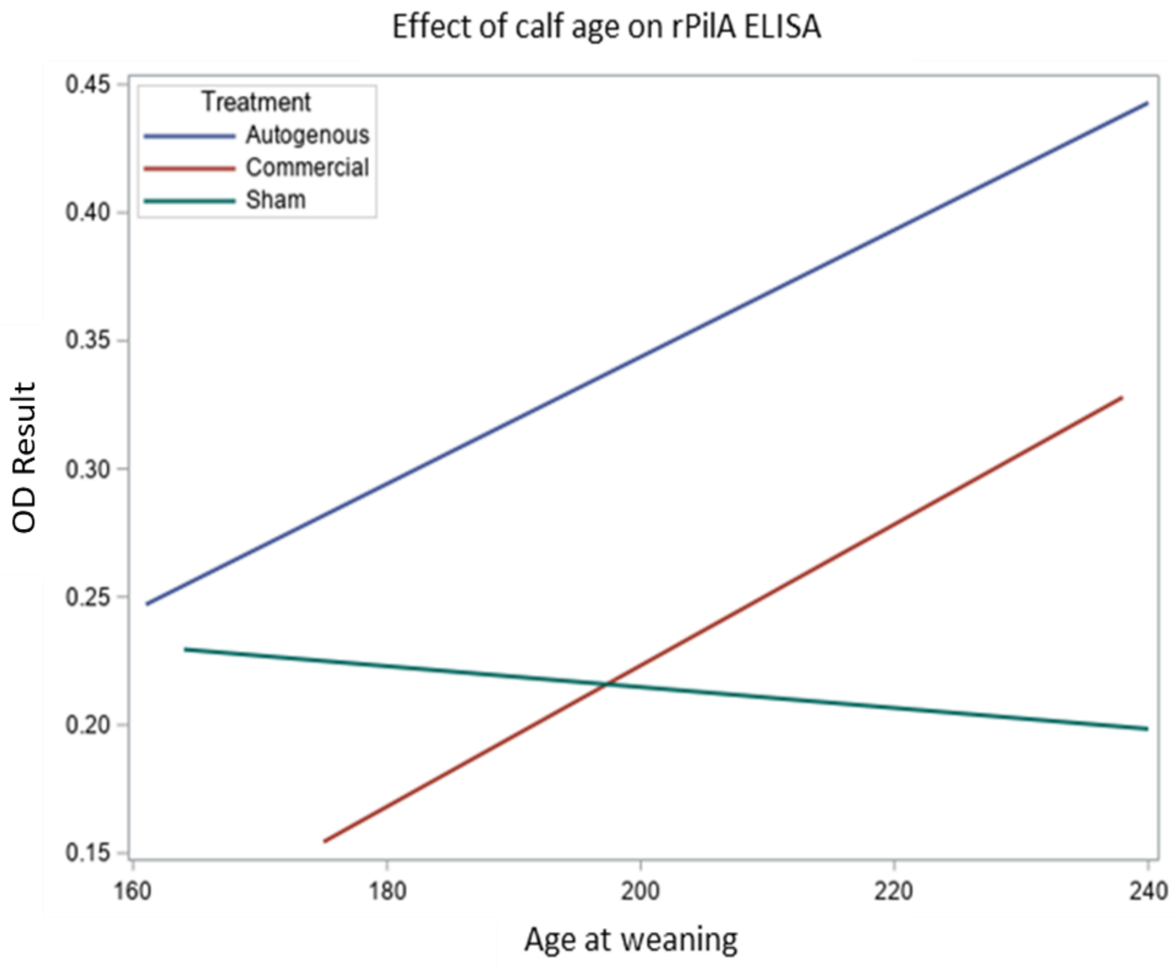

Supplementary Figure S1: ELISA OD result to the rPilA antigen by calf age and treatment. Age had an effect on antibody levels (P = 0.005).

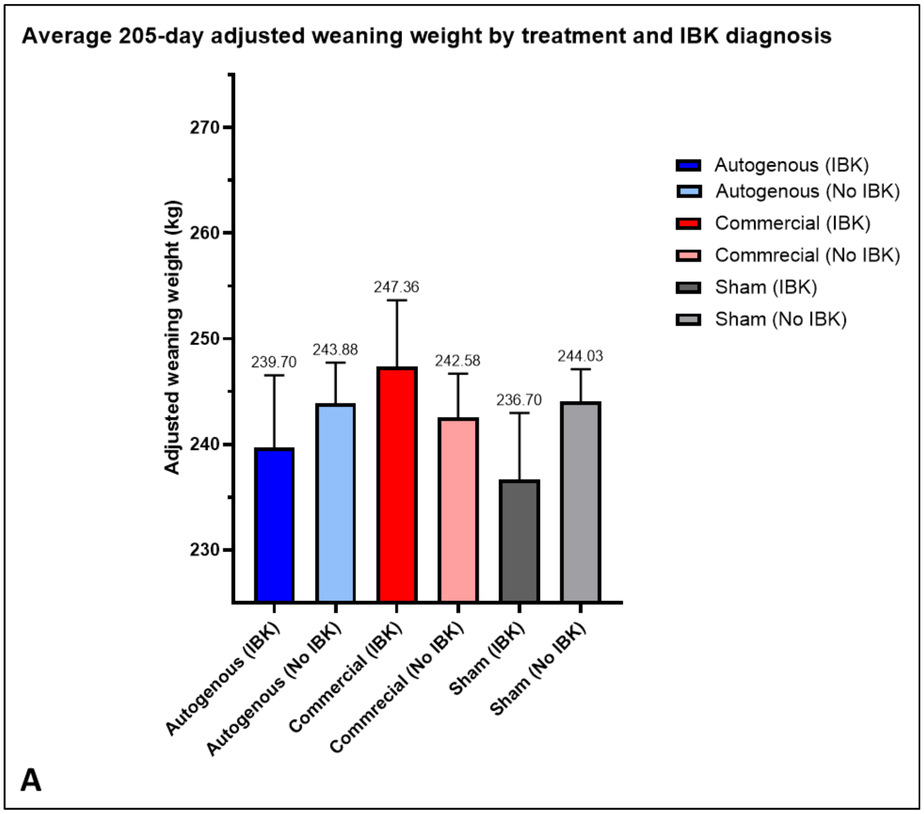

Average 205-day adjusted weaning weight by treatment and IBK diagnosis
Adjusted weaning weight (kg)
270
260
250
240
230
239.70
243.88
247.36
242.58
236.70
244.03
Autogenous (IBK)
Autogenous (No IBK)
Commercial (IBK)
Commrecial (No IBK)
Sham (IBK)
Sham (No IBK)
A

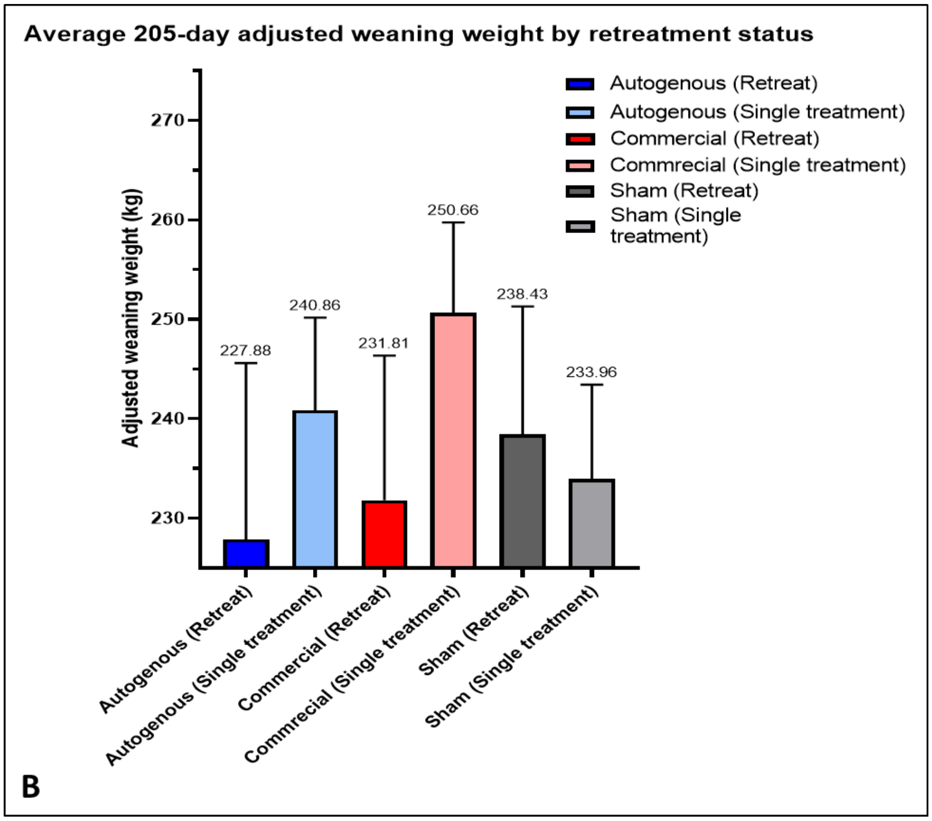

Supplementary Figure S2: Average 205-day adjusted weaning weight (in kg) by treatment group and IBK diagnosis (**A**). Autogenous: IBK: 239.70, No IBK: 243.88, Commercial: IBK: 247.36, No IBK: 242.58, Sham: IBK: 236.70, No IBK: 244.03. (IBK effect P = 0.33). Average 205 day adjusted weaning weight by treatment group and retreatment status (**B**). Autogenous: Retreatment: 227.88, Single treatment: 240.86, Commercial: Retreatment: 231.81, Single treatment: 250.66, Sham: Retreatment: 238.43, Single treatment: 233.96. (P for retreatment effect = 0.06).
